# Association between high serum Nogo-B and hypertension in Chinese Han

**DOI:** 10.1186/s12872-022-02691-w

**Published:** 2022-06-03

**Authors:** Shunuo Li, Jianmeng Zheng, Xiaoxia Dong, Shasha Bi, Liqin Duan, Wei Zheng, Peishi Yan

**Affiliations:** 1 Department of Cardiology, Dalian Municipal Central Hospital, Dalian, China; 2 Dalian Medical University, Dalian, China

**Keywords:** Blood pressure, Nogo-B, Hypertension, Risk factors

## Abstract

**Background:**

Cellular and animal studies have shown that endoplasmic reticulum protein B (Nogo-B) is associated with hypertension, but that association has not been fully studied in humans. Therefore, the expression levels of Nogo-B were investigated in hypertensive patients.

**Methods:**

The plasma Nogo-B levels of 74 patients with hypertension and 67 non-hypertensive patients were measured by enzyme-linked immunosorbent assay.

**Results:**

The plasma Nogo-B levels in the hypertensive group [523.43(411.41−746.79)] were higher than in the non-hypertensive group [380.29(281.57−462.13)] (*P* < 0.01). Pearson’s correlation analysis indicated that systolic blood pressure and diastolic blood pressure were linearly and positively correlated with plasma Nogo-B levels. Multivariable logistic regression analysis was performed based on sex, age, BMI, smoking history, drinking history, and levels of TC, TG, LDL, and HDL. The results indicated that the plasma Nogo-B levels were independently associated with hypertension (OR = 1.007, 95%CI: 1.004–1.010, *P* < 0.01).

**Conclusions:**

The present study suggests that hypertensive participants exhibited higher plasma Nogo-B levels than those without hypertension. Plasma Nogo-B levels are independently associated with hypertension.

## Introduction

Hypertension is a leading modifiable cause of cardiovascular morbidity and mortality and is highly prevalent worldwide. According to the Chinese Cardiovascular Disease Report in 2018, 290 million patients worldwide have cardiovascular diseases, of whom 245 million have hypertension [1]. The prevalence and mortality of hypertension will increase as the population ages. At present, the causes of hypertension are not clear, and its development is complex, which may be attributed to several factors, such as blood volume, vascular reactivity, neuroendocrine regulation, insufficient exercise, and gene polymorphisms [2, 3]. Although the hypertension control rates are improved by intervening on these risk factors, this condition remains an important public health challenge. In order to better prevent and control hypertension, factors associated with hypertension have to be explored.

Vascular remodeling from hypertension is an independent risk factor for mortality. The endothelial-derived relaxation factor nitric oxide (NO) [4] contributes to dilating blood vessels, inhibiting oxygen-derived free radicals, preventing white blood cell adhesion to the vascular wall, and inhibiting vascular proliferation. In vitro and in vivo studies [5] have shown that endoplasmic reticulin B (Nogo-B), which is a member of the protein family 4 (reticulon protein family 4, Rtn4) and expressed in both vascular endothelium and vascular smooth muscle cells, can negatively regulate neurite outgrowth in vascular endothelial cells, control NO synthesis through sphingosine-1-phosphate (S1P) and nitric oxide synthase (eNOS), and subsequently regulate vasoconstriction and vasodilation and, therefore, blood pressure. In addition, an in vivo study demonstrated that Nogo-B knockout mice developed hypotension at maturity and exhibited no elevated blood pressure response following stimulation with angiotensin II (AngII). These findings suggested a link between Nogo-B and hypertension. There is a lack of population-based evidence regarding the association between Nogo-B and hypertension.

Despite conclusive evidence from fundamental studies regarding the function of Nogo-B, its role in actual patients is yet to be determined. Therefore, the present study aimed to assess the association between Nogo-B and hypertension in the general Chinese population.

## Materials and methods

### Study participants

The study enrolled patients hospitalized at the Dalian Central Hospital from July 2019 to December 2020. The participants were selected by random sampling. The study was approved by the ethics committee of Dalian Central Hospital. All participants signed the informed consent form.

The selection criteria for the hypertensive patients were (i) ethnicity (Han), (ii) ≥ 18 years of age, (iii) hypertension, and (iv) no antihypertensive drugs. The selection criteria for the controls were (i) ethnicity (Han), (ii) ≥ 18 years of age, and (iii) no antihypertensive drugs. The exclusion criteria for all participants were (i) clinical suspicion of diseases that might cause secondary hypertension (such as renal artery stenosis, coarctation, glomerulonephritis, pyelonephritis, pheochromocytoma, Cushing’s syndrome, and Conn’s syndrome), (ii) coronary syndrome with coronary heart disease, including unstable angina, ST-segment elevation myocardial infarction, or myocardial infarction associated with non-ST-segment elevation, (iii) heart failure, (iv) pulmonary artery hypertension, (v) liver cirrhosis Child score grade C [6], (vi) systemic lupus erythematosus, (vii) severe liver and kidney insufficiency, (viii) therioma, (ix) pregnancy, (x) infectious disease, (xi) diabetes, or (xii) atrial fibrillation.

After excluding the patients who met the exclusion criteria, 141 participants were enrolled, with 74 new primary hypertensive patients and 67 non-hypertensive patients (control group).

### Measurement of plasma Nogo-B levels

Venous blood (2 ml) from the participants was obtained in the morning in a tube containing sodium citrate anticoagulant. The plasma was placed in an Eppendorf tube. The sample was centrifuged at 3000 r/min for 10 min and stored at −80 °C for until analysis. The plasma Nogo-B levels were determined at the Harbin Nachuan Biological Laboratory. The personnel that performed the measurements was blinded to the clinical characteristics of the study participants. The ELISA double antibody sandwich method was used to determine the plasma levels of Nogo-B. This method included a Human Nogo-B/RTN 4B ELISA kit (96 wells×2, RayBiotech Corporation, United States). All samples were processed in duplicate. A standard curve was constructed, from which the plasma Nogo-B levels of the samples were determined.

### Data collection

Demographic data, including age and sex, were obtained using questionnaires administered by trained staff. Cigarette smoking was categorized as current smoking or non-smoking. Current smoking was defined as having smoked at least 100 cigarettes in the entire lifetime, having smoked cigarettes regularly, and smoking currently. Alcohol consumption was categorized as current drinking or not. Alcohol consumption was defined as current drinkers or someone who had not consumed alcohol for a short period but had done so in the past year.

Body weight (kg) and height (cm) were measured by trained staff. The participants wore light clothes and had no shoes. Body mass index (BMI) was calculated by dividing the subjects’ weight in kilograms by their height squared in meters (kg/m^2^). Fasting glucose and blood lipid levels, including total cholesterol, triglyceride, high-density lipoprotein cholesterol (HDL-C), and low-density lipoprotein cholesterol (LDL-C), were measured by standard laboratory methods.

### Measurement of blood pressure and definition of hypertension

The blood pressure was measured using a mercury sphygmomanometer as follows. Rapid inflation was performed to increase the balloon pressure by 30 mmHg, following radial pacing. The pressure in the device was allowed to decrease at a constant rate (2 mmHg/s) slowly. If the heart rate was slower, the decrease rate followed this rate. Following the diastolic blood pressure (DBP) reading, the sphygmomanometer pressure was quickly decreased to zero. The oscillometric cuff signal was carefully assessed during the decrease, and the vertical height of the first phase (first phase) and the phase (disappear) of the mercury column were observed. The SBP and DBP readings were obtained based on the Korotkoff sounds. The blood pressure value was assessed based on the last value estimated to 0, 2, 4, 6, or 8 as the last digit, not 1, 3, 5, 7, or 9, because the graduation on the sphygmomanometer was by 2 mmHg increments. According to the Chinese Guidelines for Hypertension 2018, hypertension was defined as systolic blood pressure (SBP) ≥ 140 mmHg and/or DBP ≥ 90 mmHg (1 mmHg = 0.133 kPa), or a previous history of hypertension with SBP/DBP > 140/90 mmHg [7].

### Statistical analysis

All statistical analyses were conducted using SPSS 23.0 (IBM, Armonk, NY, USA). The continuous data that followed the normal distribution were presented as means ± standard deviation and analyzed using the t-test, whereas the data that were not distributed normally were presented as medians (range) and analyzed using the Mann-Whitney U-test. The categorical data were presented as *n* (%) and analyzed using the chi-square test. The Pearson correlation analysis was used to assess the correlation between blood pressure and Nogo-B levels for all participants. In order to analyze the correlation between blood pressure and NogoB levels, the NogoB was logarithmically transformed to normalize its distribution. In addition, a linear regression analysis of plasma Nogo-B and SBP and DBP was performed. Univariable and multivariable biclassification logistic regression analyses were performed to assess the correlation between plasma Nogo-B levels and hypertension. In addition, all participants were divided into four groups based on the quartile of plasma Nogo-B. The odds ratio (OR) of hypertension and the 95% confidence interval (CI) were calculated with the lowest quartile as a reference. The model fit was assessed using a Hosmer-Lemeshow goodness-of-fit test. In addition, the participants were divided into two groups according to the plasma Nogo-B median level: high Nogo-B (above median: 434.09 pg/ml) and lower Nogo-B (below median). The prevalence of hypertension in the high and low plasma Nogo-B groups was analyzed using binary unifactorial logistic regression with adjustment for age, sex, smoking, alcohol consumption, BMI, TC, TG, LDL-C, and HDL-C. Finally, potential covariables, such as age, sex, smoking, alcohol, BMI, and levels of TC, TG, LDL-C, and HDL-C, were included in the multifactorial models. *P* < 0.05 was considered to indicate statistically significant differences.

## Results

### Clinical characteristics

A total of 141 participants, including 71 males and 70 females, were included in the analysis. The average age was 56.5 ± 13.0 years (range: 20–90 years). There were 35 (24.8%) cigarette smokers and 29 (20.6%) alcohol consumers. The average level of the plasma Nogo-B concentration was 509.11 pg/ml (range: 139.41–1199.46 pg/ml). The characteristics of the participants are presented in Table 1. Participants with hypertension were more likely to be older, male, cigarette smokers, and alcohol consumers. In addition, higher BMI, TC, TG, and LDL-C and lower HDL-C were observed compared with those subjects without hypertension (all *P* < 0.05). Log-transformed plasma Nogo-B in hypertensive patients was significantly higher than those in patients without hypertension (*P* < 0.001).

**Table 1:** Characteristics of the study participants

Characteristics	Hypertensive subjects (*n* = 74)	Non-hypertensive subjects (*n* = 67)	*P* value
Age, years	56.6 ± 14.0	54.3 ± 11.8	< 0.05
Male	42 (59.2)	29 (33.7)	< 0.05
Smoker	22 (29.7)	13 (19.4)	< 0.05
Alochol consumer	17 (23.0)	12 (17.9)	< 0.05
BMI, kg/m^2^	25.00 ± 3.36	23.90 ± 3.52	< 0.05
SBP, mmHg	150.42 ± 12.47	121.06 ± 15.54	< 0.01
DBP, mmHg	92.95 ± 8.73	77.13 ± 8.40	< 0.01
TC, mmol/L	4.65 (3.88–5.18)	4.52 (4.04–5.29)	< 0.05
TG, mmol/L	1.98 (1.45–2.88)	1.41 (1.06–2.25)	< 0.05
LDL-C, mmol/L	2.70 (2.25–3.35)	2.68 (1.90–3.16)	< 0.05
HDL-C, mmol/L	1.14 (0.96–1.52)	1.15 (0.89–1.60)	< 0.05
Nogo-B, pg/mL	523.43(411.41-746.79)	380.29(281.57-462.13)	< 0.01
Log-transformed plasma Nogo-B, pg/mL	2.75 ± 0.19	2.55 ± 0.18	< 0.01

Results are expressed as median (interquartile range), mean ± SD or *n* (%)

*BMI* Body mass index; *SBP* Systolic blood pressure; *DBP* Diastolic blood pressure; *TC* Total cholesterol; *TG* Triglyceride; *LDL-C* Low-density lipoprotein cholesterol; *HDL-C* High-density lipoprotein cholesterol

### Correlation between plasma Nogo-B levels and blood pressure

**Fig. 1:**
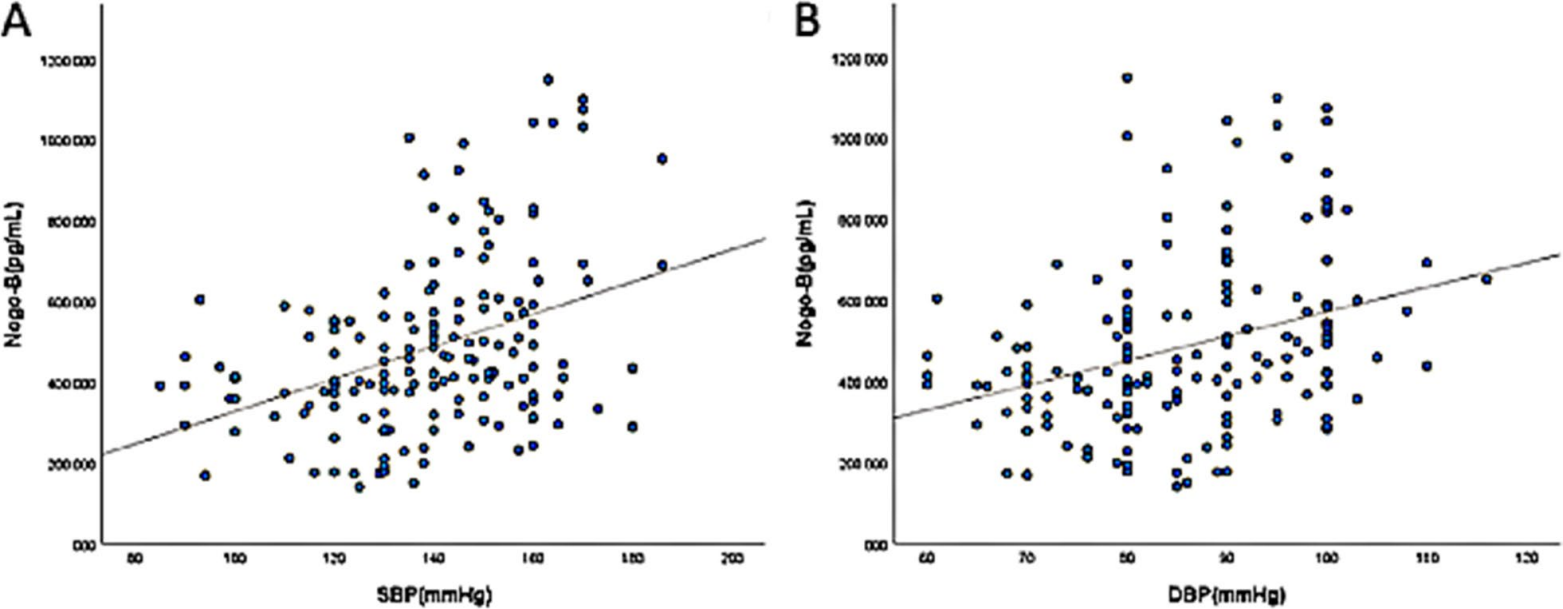
Scatterplot showing a significant and positive correlation of log-transformed plasma Nogo-B concentration levels with systolic in (**A**) (*r* = 0.395, *P* < 0.01) and diastolic in (**B**) (*r* = 0.335, *P* < 0.01) blood pressure among total participants

The Pearson correlation analysis indicated a significant linear correlation between blood pressure and plasma Nogo-B levels. As shown in Fig. 1, SBP (*r* = 0.395, *P* < 0.01) and DBP (*r* = 0.335, *P* < 0.01) correlated positively with Nogo-B levels.

### Association between plasma Nogo-B levels and hypertension

As shown in Table 2, the univariable analysis indicated a significant association between plasma Nogo-B levels and hypertension (OR = 1.006, *P* < 0.01), suggesting that for each higher 1 pg/ml plasma Nogo-B, the odds of hypertension are increased by 0.6%. When participants were divided into four groups based on the quartiles of plasma Nogo-B (the lowest quartile as the reference), the ORs in the third and quartiles were 3.85 and 22.39 (both *P* < 0.01). The *P* for trend was < 0.01.

**Table 2:** Odds ratio for the association of Nogo-B levels and hypertension

Nogo-B, pg/ml	N	Hypertension (%)	OR (95% CI)	*P* value
139.41–1099.46	141	74 (52.5)	1.006 (1.004–1.009)	< 0.0001
*Quartiles*				
139.41–355.84	35	9 (25.7)	1 (reference)	
355.85–443.12	36	14 (38.8)	1.838 (0.668–5.056)	0.238
443.13–603.64	35	20 (57.1)	3.852 (1.401–10.590)	< 0.01
603.65–3642.67	35	31 (88.5)	22.389 (6.176–81.196)	< 0.01
*P* value for trend				< 0.01

When the quartiles of plasma Nogo-B levels were included in logistic models, a Hosmer–Lemeshow goodness-of-fit test was applied to examine the model fit (X^2^ = 4.53, *P* = 0.01 for unadjusted model; X^2^ = 1.02, *P* = 0.23 for adjusted model). *CI* Confidence interval; *OR* Odds ratio

### Subgroup analysis on the association between plasma Nogo-B levels and hypertension

The median Nogo-B concentration was 434.09 pg/ml, which was used to divide the participants into the high and low plasma Nogo-B groups. As shown in Table 3, the frequency of hypertension in the high plasma Nogo-B group was higher than in the low plasma Nogo-B group in all subgroups. Compared with the low plasma Nogo-B group, high plasma Nogo-B was associated with age (both subgroups), female sex, no cigarette smoking, no alcohol consumption, BMI (both subgroups), TC (both subgroups), low TG, LDL (both subgroups), and low HDL (all *P* < 0.05).

**Table 3:** Subgroup analysis of the association between Nogo-B levels and hypertension

Subgroups	Number of participants		Hypertension (%)		OR (95% CI)	*P* value
	Low plasma Nogo-B	High plasma Nogo-B	Low plasma Nogo-B	High plasma Nogo-B		
*Age, years*						
< 59	40	37	11 (27.5)	28 (75.7)	8.202 (2.950–22.805)	< 0.01
≥ 59	31	33	12 (38.7)	23 (69.7)	3.642 (1.292–10.263)	< 0.05
*Sex*						
Male	38	32	11 (28.9)	21 (65.6)	6.562 (0.287–18.832)	0.06
Female	33	38	12 (36.4)	30 (78.9)	4.686 (1.704–12.888)	< 0.01
*Cigarette smoking*						
No	54	52	16 (29.6)	36 (69.2)	5.344 (2.331–12.248)	< 0.01
Yes	17	18	7 (41.2)	15 (83.3)	7.143 (0.484–24.384)	0.14
*Alcohol consumption*						
No	57	55	17 (29.8)	40 (72.7)	6.275 (2.761–14.261)	< 0.01
Yes	14	15	6 (42.8)	11 (73.3)	3.667 (0.771–17.429)	0.12
*Body mass index, kg/m*^2^						
< 24	34	33	8 (23.5)	22 (66.7)	6.500 (2.222–19.011)	< 0.01
≥ 24	37	37	15 (40.5)	29 (78.4)	5.317 (1.914–14.766)	< 0.01
*Total cholesterol*^a^,* mmol/l*						
< 5.18	51	53	16 (31.4)	38 (71.7)	5.542 (2.390–12.848)	< 0.01
≥ 5.18	20	17	7 (35.0)	13 (76.5)	6.036 (1.417–25.710)	< 0.01
*Triglycerides*^a^,* mmol/l*						
< 1.70	34	34	7 (20.6)	20 (58.8)	5.510 (1.879–16.159)	< 0.01
≥ 1.70	37	36	16 (43.2)	31 (86.1)	8.137 (0.585–23.619)	0.23
*Low-density lipoprotein cholesterol*^a^,* mmol/l*						
< 3.37	56	55	20 (35.7)	40 (72.7)	4.800 (2.142–10.755)	< 0.01
≥ 3.37	15	15	3 (20.0)	11 (73.3)	11.000 (1.998–60.572)	< 0.01
*High-density lipoprotein cholesterol*^a^,* mmol/l*						
< 1.04	32	26	12 (37.5)	18 (69.2)	3.750 (1.251–11.244)	< 0.01
≥ 1.04	39	44	11 (28.2)	33 (75.0)	7.636 (0.878–19.260)	0.15

Low plasma Nogo-B was defined as a value lower than the median level of plasma Nogo-B concentration, plasma Nogo-B < 434.09pg/ml; high plasma Nogo-B was defined as a value higher than the median level of plasma Nogo-B concentration, plasma Nogo-B ≥ 434.09pg/ml. In the multivariable models, confounding factors, such as age, gender, current smoking, alcohol consumption, body mass index, total cholesterol, triglycerides, low-density lipoprotein cholesterol, and high-density lipoprotein cholesterol were included unless the variable was used as a subgroup variable

*CI* Confidence interval; *OR* Odds ratio

^a^Subgroups by blood lipids were based on the recommendations from the Working Group on dyslipidemia in China: optimal and abnormal

### Multiple logistic regression analysis on the factors affecting the incidence of hypertension

As shown in Table 4, the logistic model revealed that Nogo-B levels were independently associated with hypertension (OR = 1.007, 95%CI: 1.004–1.010, *P* < 0.01) after adjust for age, sex, BMI, history of smoking, alcohol consumption, and TC, TG, HDL, and LDL.

**Table 4:** Multiple logistics regression analysis examining the factors associated with hypertension

Influence factor	OR (95% CI)	*P* value
Age	2.507 (1.633–3.586)	< 0.05
Sex	2.597 (1.570–4.472)	< 0.05
Body mass index	1.073 (1.040–1.225)	< 0.01
Cigarette smoking	2.291 (1.380–4.387)	< 0.05
Alcohol consumption	2.263 (1.370–4.315)	< 0.05
Total cholesterol	1.841 (1.519–2.508)	< 0.05
Triglycerides	1.812 (1.044–3.144)	< 0.05
Low-density lipoprotein cholesterol	1.605 (1.288–2.272)	< 0.05
High-density lipoprotein cholesterol	1.507 (1.343–3.261)	< 0.05
Nogo-B levels	1.007 (1.004–1.010)	< 0.01

*CI* Confidence interval; *OR* Odds ratio

## Discussion

In this study, we explored the correlation between plasma Nogo-B levels and hypertension. This was the first study to assess the association between human plasma Nogo-B levels and hypertension. The results indicated significantly higher plasma Nogo-B levels in patients with hypertension than in controls, whereas elevated Nogo-B levels were also independently associated with hypertension. The present study indicated that elevated plasma Nogo-B levels might be a risk factor or marker of hypertension, but the exact nature of the factor will have to be determined using longitudinal studies.

Over the past few decades, numerous studies have been performed on hypertension-associated risk factors and markers. In addition to known risk factors, such as increased weight and smoking, previous studies in recent years have found several plasma markers that may be related to hypertension. The markers hemoglobin and transferrin [8], serum furin [9, 10], unsaturated fatty acids [11], and neurohypotensin [12] may have predictive significance for the onset of hypertension. A cross-sectional study indicated that serum Corin levels were significantly higher in hypertension than in normal subjects, whereas animal experiments indicated that Corin was a type II transmembrane serine protease highly expressed in the heart that could activate natriuretic peptide and exhibit antihypertensive effects under physiological circumstances. The clinical research results were inconsistent with the animal experiments [13].

Recent fundamental studies showed that Nogo-B was able to maintain and regulate blood pressure, which was consistent with the results reported in the present study. Indeed, in 2015, Di Lorenzo et al. [14] indicated that Nogo-B-knockout mice developed hypotension and exhibited a lack of blood pressure elevation following stimulation by angiotensin II. Additional mechanistic studies indicated that Nogo-B could negatively regulate the synthesis of S1P through the S1P-eNOS-NO signaling pathway. Jozefczuk et al. [15] demonstrated that serine palmitoyltransferase (SPT), which controls S1P synthesis, could be used as a target for blood pressure intervention. Animal experiments further demonstrated the important role of the S1P-eNOS-NO signaling pathway in regulating hypertension. Heart failure, myocardial hypertrophy, and pulmonary hypertension are associated with higher Nogo-B levels [5, 14, 16]. Therefore, patients with these conditions were excluded from the present study.

The results of the multifactorial analysis indicated that high plasma Nogo-B was independently associated with hypertension, such as older age, men, overweight, smoking, and hyperlipidemia. However, in subgroups divided by sex, smoking, drinking, and TG and HDL levels, the OR values in women participants, non-smokers, non-alcoholics, and those with normal TG and HDL levels exhibited significant differences compared with those of men, drinkers, smokers, and people with abnormal TG or HDL levels. Therefore, whether Nogo-B can be used as an intervention target for preventing and treating hypertension remains to be further studied.

Nogo-B regulated NO generation through the S1P-eNOS-NO signaling axis [17–19]. These results may provide a framework for studying other pathological conditions caused by changes in Nogo-B activity [19], notably those associated with the cardiovascular system, such as hypertension, coronary heart disease [20], heart failure [16], and cardiomyopathy [17, 18]. This study excluded coronary heart disease, heart failure, tumors, and other diseases that could cause secondary hypertension. In addition to measuring plasma Nogo-B levels, the major traditional risk factors for hypertension were analyzed, such as advanced age, hyperlipidemia, hyperglycemia, alcohol consumption, and smoking. During data analysis, all routine risk factors were controlled to reduce their confounding effects and perform subgroup analysis.

Nevertheless, several limitations should be acknowledged. Firstly, it was a single-center case-control study involving only 141 patients, and the sample size was small. Further cross-sectional and cohort studies of larger sample sizes are required to confirm this conclusion. Secondly, this study could not assess the causal relationship between Nogo-B plasma concentration and the incidence of hypertension. Prospective cohort studies are required to confirm the causal relationships between these two factors. Thirdly, the diagnosis of hypertension was mainly based on the casual blood pressure, which might lead to misclassification of a part of individuals to a certain extent. Nevertheless, a standardized causal blood pressure measurement was reported to be similar to ambulatory blood pressure monitoring [21]. Fourthly, although the present study concluded that plasma Nogo-B concentration levels exhibited a linear association with blood pressure, excluding the subjects who received hypertensive treatment was a significant limitation of the study design. In addition, the failure to study the difference between Nogo-B concentration levels prior to and following the onset of hypertension is a significant limitation of the current study. Evidence regarding the half-life of Nogo-B and the optimal period for testing is unknown. Furthermore, whether blood pressure control affects Nogo-B levels, or vice-versa, is unknown and will have to be answered by future studies. Finally, this study failed to detect the eNOS activity and did not reflect Nogo-B activity.

## Conclusions

In summary, plasma Nogo-B levels are high in hypertensive subjects, and Nogo-B levels were linearly associated with SBP and DBP. However, the association between plasma Nogo-B levels and hypertension requires further research in different populations. It will facilitate further understanding of the pathogenesis of hypertension, possibly suggesting Nogo-B as a future antihypertensive target to be used for the development of novel therapeutic targets.

## Data Availability

All data generated or analysed during this study are included in this published article.

## Abbreviations

Nogo-B: Endoplasmic reticulum protein B
ELISA: Enzyme-linked immunosorbent assay
SBP: Systolic blood pressure
DBP: Diastolic blood pressure
S1P: 1-phosphate
eNOS: Nitric oxide synthase
NO: Nitric oxide
AngII: Angiotensin II
BMI: Body mass index
HDL-C: High-density lipoprotein cholesterol
LDL-C: Low-density lipoprotein cholesterol
SBP: Systolic pressure
DBP: Diastolic pressure
SPT: Serine palmitoyltransferase
